# Real‐World Treatment Patterns and Physician Perspectives in Relapsed/Refractory Multiple Myeloma: Results From a Nationwide Italian GIMEMA Survey

**DOI:** 10.1002/jha2.70354

**Published:** 2026-07-25

**Authors:** Francesca Fazio, Alfonso Piciocchi, Paolo de Fabritiis, Messina Messina, Stefano Soddu, Valeria Sargentini, Francesco Di Raimondo, Francesca Patriarca, Pellegrino Musto, Maurizio Offidani, Elena Zamagni, Sara Bringhen, Massimo Gentile, Giuseppe Mele, Paola Fazi, MariaTeresa Petrucci, Michele Cavo

**Affiliations:** ^1^ Hematology Department of Translational and Precision Medicine Sapienza University of Rome‐Azienda Policlinico Umberto I Rome Italy; ^2^ GIMEMA Foundation Rome Italy; ^3^ Hematology Unit and BMT AOU Policlinico G. Rodolico ‐ S. Marco Università di Catania Catania Italy; ^4^ Hematology and Transplant Center Unit Azienda Sanitaria Universitaria Friuli Centrale DMED Udine University Udine Italy; ^5^ Unit of Hematology and Stem Cell Transplantation AOUC Policlinico Bari “Aldo Moro” University School of Medicine Department of Precision and Regenerative Medicine and Ionian Area Bari Italy; ^6^ Clinica Di Ematologia AOU delle Marche Ancona Italy; ^7^ Department of Medical and Surgical Sciences University of Bologna Bologna Italy; ^8^ Dipartimento Di Scienze Mediche e Chirurgiche Università di Bologna Bologna Italy; ^9^ Hematology ASO Santa Croce e Carle di Cuneo Cuneo Italy; ^10^ Hematology Unit, Azienda Ospedaliera Annunziata Cosenza Italy; ^11^ Department of Pharmacy Health and Nutritional Sciences University of Calabria Rende Italy; ^12^ U.O.C. di Ematologia‐Unità Trapianto di Midollo Osseo PO Antonio Perrino Brindisi Italy

**Keywords:** multiple myeloma, real‐world, relapsed/refractory multiple myeloma

## Abstract

**Background:**

The management of relapsed/refractory multiple myeloma (RRMM) remains complex due to early exposure to multiple drug classes since the first treatment line.

**Aims:**

To evaluate treatment patterns and identify unmet clinical needs in RRMM across Italian hematology centers.

**Methods:**

A national survey was conducted among hematologists experienced in MM care and working in 71 centers, collecting aggregated data on treatment lines, regimens, and clinician perceptions.

**Results:**

Upfront daratumumab‐based regimens were used most frequently. Lenalidomide and anti‐CD38 refractoriness emerged early, limiting options in 2L/3L. Anti‐BCMA therapies were broadly endorsed.

**Conclusions:**

Despite therapeutic advances for MM, real‐world data reveal significant treatment variability and reinforce the need for shared guidelines and innovative therapies.

**Trial Registration**: The authors have confirmed clinical trial registration is not needed for this submission

## 1 Introduction

Multiple myeloma (MM) is a heterogeneous hematologic malignancy whose treatment landscape has evolved substantially over the last decade, particularly with the introduction of monoclonal antibodies, next‐generation immunomodulatory drugs (IMiDs) and proteasome inhibitors (PIs). These advances have extended survival but raised new challenges. In first‐line (1L) therapy, especially, the combination of anti‐CD38 monoclonal antibodies with IMiDs and PIs to form quadruplet regimens has significantly modified standard practice, yet most patients eventually relapse and become refractory to previously used drug classes [1]. Therefore, the management of patients with relapsed/refractory MM (RRMM) who have received one or more prior lines of therapy (LOTs) remains particularly complex [2, 3, 4]. The choice of therapy depends on expected efficacy and tolerability, duration of response (DoR) to previous regimens, number of prior LOT and prior exposure or refractoriness to specific drug classes, including IMiDs, PIs, anti‐CD38 and ‐SLAM7S monoclonal antibodies, and emerging agents such as immune therapies targeting BCMA.

Real‐world treatment patterns, patient distribution across LOTs, and emerging unmet clinical needs are essential to guide optimal treatment strategies. To address these issues, the GIMEMA (Gruppo Italiano Malattie Ematologiche dell'Adulto) MM Working Group conducted a national survey that was sent to all GIMEMA centers involved in MM care.

## 2 Methods

Between February and March 2025, a national cross‐sectional survey involving 120 hematological centers distributed across the country was conducted. The present analysis is based on the 71 centers that responded to the survey with complete data. These centers are distributed across the entire Italian territory (35 centers in northern Italy; 19 centers in central Italy and 17 centers in southern Italy), allowing a broad nationwide overview of treatment patterns in routine clinical practice. A complete list of the investigators and participating centers is reported in Table S1.

The survey collected information on MM patient volumes, distribution across treatment lines (first, second, and third), clinical status (fit/frail), therapeutic regimens, exposure, and refractoriness to lenalidomide and anti‐CD38 monoclonal antibodies, specifically referring to clinical activities and treatment patterns observed during the year 2024. The questionnaire also gathered physician perspectives on retreatment strategies and novel agents such as anti‐BCMA therapies.

The survey collected structured treatment information for 1L, second‐line (2L), and third‐line (3L) therapy. In the 1L setting, detailed regimen‐level information was collected for ASCT‐ineligible patients. For ASCT‐eligible patients, the survey captured overall eligibility to receive ASCT and post‐ASCT lenalidomide maintenance but did not collect structured data on induction regimens.

Data collection was conducted using the Research Electronic Data Capture [5] (REDCap) platform, a secure web‐based application hosted on GIMEMA's institutional servers. REDCap ensured data integrity, compliance with privacy standards, and traceability of entries.

All data were aggregated to preserve patient anonymity. Descriptive statistical analyses were performed using the R software environment (version 4.3.1). Frequencies and percentages were calculated for categorical variables, while cross‐tabulations were used to explore treatment distributions and clinical patterns across centers. Inferential analyses were not conducted, as the study aimed to provide a descriptive overview of real‐world practice rather than test predefined hypotheses.

## 3 Results

### 3.1 Overall Patient Distribution

Across all centers, a total of 14,966 MM patients were followed in 2024. Of these, 3662 patients were newly diagnosed, 2776 were receiving 2L therapy and 1310 were on 3L therapy, while the remaining patients were distributed across later LOTs (Table 1). Among newly diagnosed patients, 81.7% required immediate therapy and started 1L treatment.

**TABLE 1:** Overall MM population.

**Category**	***N***	**%**
Total MM patients followed	14,966	—
Newly diagnosed (ND)	3662	24.5%
Patients in 2L	2776	18.5%
Patients in 3L	1310	8.8%
Patients in >3L	7218	48.2%

### 3.2 First‐Line Therapy

Among the 2992 patients who initiated 1L therapy, 1743 (58.3%) were considered ineligible for autologous stem cell transplantation (ASCT). In this population, daratumumab‐based regimens were prevalent, accounting for 1307 patients: 67.2% received Dara‐Rd (daratumumab/lenalidomide/dexamethasone) and 8.1% received Dara‐VMP (daratumumab/bortezomib/melphalan/dexamethasone). Daratumumab‐sparing regimens included Rd (lenalidomide/dexamethasone; *n* = 312, 18.0%), VRd (bortezomib/lenalidomide/dexamethasone; *n* = 50, 2.9%) and VMP (bortezomib/melphalan/prednisone; *n* = 23, 1.3%). Lenalidomide was also widely used post‐ASCT: 2653 patients received lenalidomide maintenance in 2024. A summary of 1L characteristics and treatment distribution is presented in Table 2.

**TABLE 2:** Overview of multiple myeloma patient distribution and treatment patterns in 2024: First‐line (1L) treatment.

**Category**	**Variable**	**Year of AIFA approval**	***N***	**%**
New diagnoses	Patients starting 1L therapy		2992	81.7%
Eligibility	ASCT‐eligible		1249	41.7%
	ASCT‐ineligible		1743	58.3%
1L regimens (ASCT‐ineligible)	Dara‐Rd	2021	1167	67.2%
	Dara‐VMP	2021	140	8.1%
	Rd	2016	312	18.0%
	VRd	2021	50	2.9%
	VMP	2018	23	1.3%
	Other		45	2.6%
Post‐ASCT	Lenalidomide maintenance		2653	—

### 3.3 Second‐Line Therapy

Among the 2776 patients receiving 2L treatment, 67.4% were classified as fit and 32.6% as frail. Most patients (89.4%) remained on 2L therapy during the survey period.

Among the overall 2L population, 747 patients (approximately 27% of all RRMM patients receiving 2L therapy) were lenalidomide‐refractory. Regarding anti‐CD38 exposure, 447 patients were anti‐CD38 refractory, while 493 were anti‐CD38 naive after one prior line.

Treatment patterns reflected both refractoriness and clinical preference. Among the 2776 patients, treatment data were available for 2384. The most frequently used regimen was Dara‐Rd (daratumumab/lenalidomide/dexamethasone; 887 patients, 37.2%).

In lenalidomide‐refractory patients, commonly used regimens included IsaKd (isatuximab/carfilzomib/dexamethasone) (437 patients, 18.3%), DPd (daratumumab/pomalidomide/dexamethasone) (225 patients, 9.4%) and DVd (daratumumab/bortezomib/dexamethasone) (134 patients, 5.6%). These figures reflect the use of these combinations within the overall 2L‐treated population, as these regimens are also employed in patients who are lenalidomide‐exposed but not refractory or lenalidomide‐naive.

In the 2L setting, PI‐based regimens such as SVd (selinexor/bortezomib/dexamethasone), PVd (pomalidomide/bortezomib/dexamethasone) or Kd (carfilzomib/dexamethasone) represented relevant treatment options, particularly in patients with prior lenalidomide refractoriness: PVd (143 patients, 6%; pomalidomide/bortezomib/dexamethasone), SVd (100 patients, 4.2%; selinexor/bortezomib/dexamethasone) and Kd (95 patients, 4%; carfilzomib/dexamethasone). Details on 2L patient profiles, refractoriness, and treatment patterns are reported in Table 3.

**TABLE 3:** Overview of multiple myeloma patient distribution and treatment patterns in 2024: Second‐line (2L) treatment.

**Category**	**Variable**	**Year of AIFA approval**	***N***	**%**
Population	Total 2L patients		2776	18.5%
	Treatment data available		2384	85.9%
Clinical status	Fit		1872	67.4%
	Frail		904	32.6%
Refractoriness / exposure	Lenalidomide‐refractory		747	26.9%
	Lenalidomide‐exposed, not refractory		165	5.9%
	Anti‐CD38 naïve		493	17.8%
	Anti‐CD38 refractory		447	16.1%
Main 2L regimens	DRd	2017	887	37.2%
	Isa‐Kd	2022	437	18.3%
	KRd	2016	161	6.8%
	DPd	2023	225	9.4%
	DVd	2020	134	5.6%
	PVd	2020	142	6.0%
	SVd	2024	101	4.2%
	Kd	20216	95	4.0%
	Other		202	8.5%

### 3.4 Third‐Line Therapy

A total of 1310 patients received 3L treatment (59% fit and 41% frail), with treatment data available for 1109 patients. In 3L setting, we recorded an increased use of SVd, which increased markedly: 244 patients (22%) received the novel XPO1‐inhibitor selinexor‐containing regimens. Anti‐CD38‐based regimens (primarily isatuximab‐based combinations) were used in 247 (22.3%) patients: IsaPd (isatuximab/pomalidomide/dexamethasone, 9.5%); IsaKd (3.4%) and EloPd (elotuxumab/pomalidomide/dexamethasone, 9.4%). PI‐based regimens were used in 257 (23.2%) patients: Kd (12.7%), KRd (carfilzomib/lenalidomide/dexamethasone) (5.5%), and PVd (5%). Daratumumab‐based regimens were less common (182 patients, 16.4%): DRd (6.8%), DPd (3.7%), and DVd (6%). 3L regimens and clinical characteristics are shown in Table 4.

**TABLE 4:** Overview of Multiple Myeloma Patient Distribution and Treatment Patterns in 2024: third‐line (3L) treatment.

**Category**	**Variable**	**Year of AIFA approval**	***N***	**%**
Population	Total 3L patients		1310	8.8%
	Treatment data available		1109	84.7%
	Fit		773	59.0%
	Frail		537	41.0%
Main 3L regimens	SVd	2024	244	22%
	Isa‐Pd	2021	105	9.5%
	Elo‐Pd	2020	104	9.4%
	Isa‐Kd	2022	38	3.4%
	DRd	2017	75	6.8%
	DPd	2023	41	3.7%
	DVd	2020	66	6%
	Kd	2016	141	12.7%
	KRd	2016	61	5.5%
	PVd	2020	55	5%
	Other		179	16.1%

Abbreviation: AIFA: Agenzia Italiana del Farmaco.

### 3.5 Unmet Medical Needs and Physician Insights

The survey included structured questions to capture clinicians’ approaches and perceptions of clinical issues. When asked whether patients exposed, but not refractory, to lenalidomide after 1L should receive lenalidomide‐based therapy in 2L, 62% responded affirmatively, supporting selective reuse under physician medical decisions.

Opinions on anti‐CD38 retreatment were divided: 52% supported its use in appropriate clinical scenarios (non‐refractoriness, sufficient treatment‐free interval), while 48% did not. Eligible patients were thought to include: (1) those who did not progress during Dara‐VTd induction and/or who relapsed during posttransplant maintenance with lenalidomide and (2) those who discontinued anti‐CD38 therapy for reasons other than refractoriness and relapse. Many centers favored a preferred washout period of > 12 months to allow potential resensitization.

All 71 centers (100%) recognized anti‐BCMA T‐cell redirecting therapies as essential for RRMM management. Regarding optimal timing, 45.1% preferred their use in 2L and 51.7% in 3L, while only 4.2% favored later lines, highlighting growing interest in earlier integration once access becomes feasible.

Progression‐free survival (PFS) was the most valued endpoint for assessing 2L treatment efficacy (57.7%), followed by MRD negativity (25.4%). Overall survival (OS) and DoR were considered less critical, reflecting a focus on durable disease control.

## 4 Discussion

The GIMEMA survey provides a comprehensive snapshot of MM care in Italy, documenting rapid adoption of monoclonal antibodies and increasing therapeutic sophistication in both 1L and RR settings. Dara‐Rd has emerged as the dominant frontline regimen in patients not eligible for transplant, in alignment with international practice guidelines.

The substantial adoption of lenalidomide maintenance in our cohort underscores its consolidated role in sustaining disease control after ASCT.

However, early onset of lenalidomide and anti‐CD38 refractoriness in a large subset of patients underscores the need for therapeutic options relying on other mechanisms of action. Importantly, the survey captures treatment information on many MM patients, representing one of the largest real‐world cohorts analyzed to date in Italy. This extensive population base provides substantial robustness and external validity to the observed treatment patterns, ensuring that the findings accurately reflect nationwide clinical practice and are not limited to isolated center experiences.

The survey captured information on lenalidomide and anti‐CD38 exposure/refractoriness, but the aggregate design did not allow complete cross‐stratified analyses of 2L regimens according to naïve, exposed, and refractory subgroups for each agent class.

Triplet regimens were frequently used in 2L and 3L, although their selection varied widely, reflecting heterogeneous patient characteristics and limited consensus regarding optimal sequencing beyond 1L. The growing use of pomalidomide and isatuximab‐based therapies suggests a shift toward next‐generation approaches, while inconsistent retreatment practices demonstrate the need for clearer evidence and guidance.

Comparable experiences have been reported internationally. In 2023, Ribbands et al. assessed physicians' approaches to MM treatment across LOTs in the United States, with 63 physicians reporting data on 377 patients [6]. Similarly, Martínez‐López et al. conducted a European survey involving 173 physicians and 2179 patients [7].

Survey responses also demonstrated strong support for early use of anti‐BCMA therapies, with over 96% of centers recommending their deployment by 3L. However, access constraints and absence of robust head‐to‐head data currently limit broader implementation.

Our findings should also be interpreted in the context of previously published real‐world experiences, including Italian studies documenting the progressive evolution of MM treatment strategies and outcomes in routine practice [8, 9, 10, 11]. These reports support the view that treatment sequencing has become increasingly complex in the era of early exposure to lenalidomide and anti‐CD38 monoclonal antibodies.

An important finding of this survey is that treatment selection in routine practice is influenced not only by disease‐ and patient‐related variables, but also by physician preference. In a therapeutic landscape where several active combinations coexist and sequencing is not fully standardized, clinicians often balance refractoriness patterns, treatment history, frailty, route of administration, expected tolerability, treatment burden, and local familiarity with specific regimens. The variability observed across centers likely reflects this multidimensional decision‐making process.

Beyond describing treatment patterns, our survey may also contribute to shared decision making and future guideline development. By identifying areas of broad agreement and areas of uncertainty, such as anti‐CD38 retreatment and treatment choice after lenalidomide exposure or refractoriness, as well as patients that could benefit from drugs with novel mechanisms of action. The survey highlights where harmonized recommendations are most needed. In these scenarios, treatment discussions increasingly require balancing efficacy expectations, toxicity profile, logistics, treatment burden, and patient preferences.

## 5 Conclusion

These preliminary results from the GIMEMA survey capture the ongoing transformation of MM care in Italy, marked by expanded use of novel agents and persistent challenges in RRMM management. Notably, the inclusion of nearly 15,000 patients provides a highly robust and nationally representative dataset, reinforcing the validity and generalizability of the reported trends. Although therapeutic options are broadening, clinical decisions remain highly individualized, and retreatment strategies lack standardization. In line with the most recent European guidelines [12], the therapeutic landscape of RRMM is expected to further evolve with earlier and more structured integration of immune‐based strategies, including CAR‐T cell therapies, bispecific antibodies targeting BCMA and alternative antigens such as GPRC5D, as well as antibody‐drug conjugates, potentially redefining treatment sequencing in the coming years. Future efforts should focus on generating comparative effectiveness data, developing consensus guidelines, and ensuring equitable access to cutting‐edge therapies, particularly those targeting BCMA. These survey‐based results capture the ongoing evolution of MM care in Italy within the GIMEMA network, marked by broad use of novel agents and increasing complexity in relapsed/refractory disease. Although treatment options are expanding, clinical decisions remain heterogeneous and retreatment strategies are not fully standardized. These findings may support future comparative analyses, consensus efforts, and broader integration of innovative immune‐based therapies in routine practice.

## Author Contributions

**Francesca Fazio**: conceptualization, study design, questionnaire development, interpretation of data, writing – original draft. **Alfonso Piciocchi**: conceptualization, study design, questionnaire development, interpretation of data, writing – original draft. **Valeria Sargentini**: data collection, formal analysis. **Stefano Soddu**: data collection, formal analysis. **Messina Messina**: data collection, formal analysis, interpretation of data, writing – original draft. **Paolo de Fabritiis**: interpretation of data, writing – original draft. **Maria Teresa Petrucci**: interpretation of data, writing – original draft. **Michele Cavo**: interpretation of data, writing – original draft. All authors contributed to the interpretation of the results and approved the final manuscript.

## Funding

This study is funded from GIMEMA (Gruppo Italiano Malattie Ematologiche dell'Adulto).

## Ethics Statement

The authors have nothing to report.

## Consent

The authors have nothing to report.

## Conflicts of Interest

Francesca Fazio: advisory board for GlaxoSmithKline; Sanofi; honoraria for Amgen, Janssen‐Cilag, GlaxoSmithKline, Sanofi; Menarini‐Stemline; Bristol‐Myers Squibb‐Celgene. Alfonso Piciocchi: advisory board: Astellas, Servier; consultant: GlaxoSmithKline, Amgen, Janssen. Francesco Di Raimondo: honoraria and advisory board for Abbie, Amgen, AstraZeneca, Bristol‐Myers Squibb, GlaxoSmithKline, Incyte, Jazz, Novartis, Pfizer, Roche, Sanofi, Lilly. Francesca Patriarca: honoraria and advisory board for GlaxoSmithKline, Amgen, Janssen, Bristol‐Myers Squibb‐Celgene, Menarini, Pfizer. Pellegrino Musto: honoraria and advisory boards for AbbVie, Amgen, Alexion, Astellas, AstraZeneca, BeiGene, Bristol‐MyersSquibb, Daichi‐Sankyo, Eli‐Lilly, Gilead, GlaxoSmithKline, Grifols, Incyte, Johnson & Johnson, Jazz, Kyowa Kirin, Menarini‐Stemline, Otsuka, Novartis, Pfizer, Roche, Sanofi, Servier, Sobi, and Takeda. Massimo Offidani: honoraria and advisory boards for Amgen, Janssen‐Cilag, Sanofi, GlaxoSmithKline, Oncopeptides, Pfizer, Menarini‐Stemline. Elena Zamagni: honoraria and advisory board for Janssen, Bristol‐Myers Squibb, Sanofi, Amgen, GlaxoSmithKline, Pfizer, Oncopeptides, Menarini‐Stemline. Sara Bringhen: honoraria for Amgen, Celgene Bristol‐Myers Squibb, GlaxoSmithKline, Janssen, Sanofi, AbbVie; advisory board for Celgene, Bristol‐Myers Squibb, Janssen, Takeda, Pfizer, GlaxoSmithKline, Menarini‐Stemline, Oncopeptides; consultancy for Sanofi. Giuseppe Mele: honoraria and advisory board for Amgen, Janssen, GlaxoSmithKline, Menarini‐Stemline, Pfizer. Maria Teresa Petrucci: Honoraria e advisory board: JJ, BMS, GSK, Pfizer, Oncopeptide, Amgen, Menarini, AbbVie, Takeda, Sanofi. Michele Cavo: consultant and/or advisory committees for Janssen, Celgene, BMS, Sanofi, Amgen, Pfizer, and Menarini Stemline, and honoraria from Janssen, Celgene, BMS, Sanofi, Amgen, Pfizer, and Menarini Stemline. The other authors declare no conflicts of interest.

## Supporting information




**Supporting Information**: jha270354‐sup‐0001‐SuppMat.docx

## Data Availability

The data that support the findings of this study are available on request from the author Alfonso Piciocchi.
