# Supplementary material for: Real‐World Treatment Patterns and Physician Perspectives in Relapsed/Refractory Multiple Myeloma: Results From a Nationwide Italian GIMEMA Survey
Source: EJHaem. 2026 Jul 25;7(4):e70354. doi: 10.1002/jha2.70354 (PMC13401147; doi:10.1002/jha2.70354)
Supplement: Supplementary file 1 — Supporting Information: jha270354‐sup‐0001‐SuppMat.docx [file JHA2-7-e70354-s001.docx]

**Supplementary Material**

**Table 1. List of Investigators and Participating Centers in the GIMEMA Multiple Myeloma Survey (N = 71)**

| **Investigator** | **Center / Institution** |
| --- | --- |
| Allegra Alessandro | AOU Policlinico G. Martino – Messina |
| Amato Gabriella | Istituto Oncologico del Mediterraneo - Catania |
| Angelucci Emanuele | IRCCS Policlinico San Martino - Genova |
| Annibali Ombretta | Campus Bio Medico - Roma |
| Antolino Giusy | AOU Sant’Andrea - Roma |
| Antonioli Elisabetta | AOU Careggi - Firenze |
| Belotti Angelo | ASST Spedali Civili - Brescia |
| Bolli Niccolò | IRCCS Istituto Nazionale Tumori – Milano |
| Bongarzoni Velia | Ospedale S. Giovanni Addolorata - Roma |
| Capochiani Enrico | AUSL Toscana Nord Ovest, Ospedale - Livorno |
| Cellini Alessandro | Unità di Ematologia e Immunologia Clinica - Padova |
| Cilloni Daniela | Ospedale Mauriziano, Umberto I - Torino |
| Conticello Concetta | Policlinico Vittorio Emanuele, PO “Rodolico” – Catania |
| Coppetelli Ugo | P.O. Santa Maria Goretti - Latina |
| Coscia Marta | ASST dei Sette Laghi - Varese |
| Cuneo Antonio | AOU Arcispedale S. Anna – Cona - Ferrara |
| Deambrogi Clara | AOU Maggiore della Carità - Novara |
| Ferraro Silvia | Ospedale S. Eugenio - Roma |
| De Padua Laura | Ospedale Fabrizio Spaziani - Frosinone |
| De Paoli Lorenzo | Ospedale S. Andrea - Vercelli |
| Derudas Daniele | AOU Brotzu – PO Businco - Cagliari |
| Di Bona Eros | AULSS 7 Pedemontana, Ospedale - Bassano del Grappa |
| Fazio Francesca | Policlinico Umberto I, Roma |
| Furlan Anna | ULSS N.2 Marca Trevignana - Treviso |
| Galimberti Sara | AOU Pisana - Pisa |
| Galli Monica | ASST Ospedale Papa Giovanni XXIII – Bergamo |
| Gamberi Barbara | Arcispedale S. Maria Nuova, IRCCS – Reggio Emilia |
| Garzia Maria Grazia | San Camillo Forlanini - Roma |
| Gentile Massimo | P.O. Annunziata – A.O. Cosenza |
| Giuliani Nicola | Azienda Ospedaliero Universitaria - Parma |
| Gottardi Michele | Istituto Oncologico Veneto - Castelfranco Veneto |
| Gozzetti Alessandro | AOU Senese - Siena |
| Krampera Mauro | AOU Policlinico G.B. Rossi - Verona |
| Laszlo Daniele | Istituto Clinico Humanitas Gavazzeni - Bergamo |
| Lazzaro Antonio | ASL Ospedale Guglielmo da Saliceto - Piacenza |
| Liberatore Carmine | P.O. Santo Spirito – Pescara |
| Maggi Alessandro | Ospedale SS. Annunziata - Taranto |
| Malerba Laura | AO Ospedali Riuniti Marche Nord - Pesaro |
| Mangiacavalli Silvia | Fondazione IRCCS Policlinico San Matteo – Pavia |
| Mannina Donato | P.O. Papardo - Messina |
| Marasco Vincenzo | Istituto Nazionale Tumori - Milano |
| Marcatti Magda | IRCCS San Raffaele |
| Marchetti Monia | Ospedali Riuniti P.O. Cardinal G. Massaia - Asti |
| Mele Giuseppe | Ospedale Perrino - Brindisi |
| Mengarelli Andrea | IFO - Roma |
| Michieli Maria Grazia | IRCCS Centro Oncologico - Aviano |
| Monaco Federico | AON SS. Antonio Biagio Cesare Arrigo - Alessandria |
| Montefusco Vittorio | ASST Santi Paolo e Carlo - Milano |
| Musso Maurizio | Casa di cura la Maddalena - Palermo |
| Musto Pellegrino | AOU Policlinico - Bari |
| Nunziata Giuseppe Rodolfo | P.O. S.G. Moscati - Aversa |
| Offidani Massimo | AOU Ospedali Riuniti - Ancona |
| Pane Fabrizio | AOU Federico II - Napoli |
| Pavan Laura | Azienda Ospedaliero Universitaria - Padova |
| Pini Massimo | ASL San Giovanni Bosco - Torino |
| Pizzuti Michele | Madonna delle Grazie - Matera |
| Podda Luigi | AOU, Cliniche Universitarie - Sassari |
| Rago Angela | Ospedale Santo Spirito - Roma |
| Rossi Elena | Policlinico Universitario Gemelli, IRCCS - Roma |
| Rossini Bernardo | IRCCS Oncologico Ist. Tumori Giovanni Paolo II - Bari |
| Russo Domenico | ASST Spedali Civili - Brescia |
| Sartori Roberto | ULSS 6 Euganea Ospedale - Camposampiero (PD) |
| Selleri Carmine | AOU S.G. di Dio e Ruggi d’Aragona - Salerno |
| Tarantini Giuseppe | Ospedale Mons. Dimiccoli - Barletta |
| Tosetto Alberto | AULSS 8 Berica, Ospedale - Vicenza |
| Turrini Mauro | Ospedale Valduce - Como |
| Vassallo Francesco | ASO S. Croce e Carle - Cuneo |
| Venditti Adriano | AOU Policlinico Tor Vergata - Roma |
| Vitolo Umberto | Istituto Fondazione per l’Oncologia - Candiolo (TO) |
| Zaja Francesco | ASUGI - Trieste |
| Zamagni Elena | IRCCS AOU, Policlinico S. Orsola – Bologna |
